# Barriers to vaccine acceptance in the adult population of mainland Finland, 2021

**DOI:** 10.1017/S0950268824000463

**Published:** 2024-03-15

**Authors:** Mervi Lasander, Kimmo Elo, Katja Joronen, Timothée Dub

**Affiliations:** 1 Infection Disease Control and Vaccinations Unit, Department of Health Security, Finnish Institute for Health and Welfare, Helsinki, Finland; 2 Faculty of Medicine, Department of Nursing Science, University of Turku, Turku, Finland; 3 Faculty of Social Sciences, Centre for Parliamentary Studies, University of Turku, Turku, Finland

**Keywords:** vaccine acceptance, vaccine communication, vaccine hesitancy, vaccine information, public opinion, survey

## Abstract

There has been a lack of information on vaccine acceptance for Finnish adults. We conducted a secondary analysis of cross-sectional data collected through the Finnish Medicines Agency Medicine Barometer 2021 survey (response rate: 20.6%). We described and explained vaccine acceptance by investigating the associations between socio-demographic factors and statements using logistic regression and conducted a factor analysis. The majority of respondents (*n* = 2081) considered vaccines to be safe (93%), effective (97%), and important (95%). However, 20% and 14% felt they did not have enough information about vaccines and vaccine-preventable diseases (VPDs), respectively. Respondents aged 18–39 were 2.8 times more likely to disagree that they had enough information about VPDs compared to respondents aged 60–79 (*p* < 0.001), while respondents with poorer self-perceived health were 1.8 times more likely to declare not having enough information about vaccines (*p* < 0.001). We generated three-factor dimensions from the eight statements. They were related to ‘Confidence and attitudes towards vaccines’, ‘Access to information on vaccines and VPDs’, and ‘Debate on vaccine issues’, which may reflect the underlying thinking patterns. Access to and understanding of information about vaccines and VPDs need to be improved for Finnish adults to increase vaccine acceptance and uptake, thus preventing the spread of VPDs.

## Introduction

Vaccine hesitancy is defined as a ‘delay in acceptance or refusal of vaccines despite availability of vaccination services’. It is complex, due to a wide range of ideological and practical factors, such as lack of information, trust in healthcare providers, and previous experiences, and varies across time [1–8]. An increased level of vaccine hesitancy will reduce vaccine acceptance and coverage, posing a major health risk to millions of people and increasing the risk of spreading vaccine-preventable diseases (VPDs) [9, 10]. Hence, the World Health Organization has listed vaccine hesitancy as one of the top ten global health threats in 2019 [11].

In 2020, vaccine acceptance in European countries was influenced by mistrust in the safety and efficacy of vaccines [7], perceived risk of communicable disease, and socio-demographic determinants or specific population groups [1, 5–8, 12–16]. Further barriers to vaccine acceptance were ideological reasons, lack of institutional confidence and confidence among healthcare workers (HCWs) [17–21], public perceptions of specific vaccines, inconvenience of vaccination services, and religious reasons or groups [7, 21]. In Finland, official websites were widely used to increase vaccination coverage, informing citizens about vaccines and their safety. Some information on the National Immunization Programme (NIP) was provided through brochures and campaigns, mainly for *human papillomavirus (HPV) and varicella zoster virus* vaccines for children. Collaboration between the government and vaccine research institutes on vaccination issues was still limited in Finland. In addition, general public information, for example in newspapers and on television, was limited. HCWs had access to information on vaccine safety through telephone counselling and training provided by the Finnish Institute for Health and Welfare, while social media and brochures were not used. Patients were informed about *influenza* and *pneumococcal* vaccinations through clinics held by HCWs [21].

In the 2019 Eurobarometer, Finns rated the efficacy and importance of vaccines at 97% and 95%, respectively, among the highest in Europe [22]. Similarly, in the 2022 Eurobarometer, Finns’ confidence in the safety of vaccines was among the highest in Europe at 87% [23]. In 2020, a Finnish survey on *severe acute respiratory syndrome (SARS-CoV-2)* vaccines found that 81% of respondents considered vaccination to be a good way to prevent disease in general [12]. In the 2022 Eurobarometer, 40% of Finns trusted health authorities as a source of reliable information, while the European average was 12%. Conversely, on average, 65% of Europeans trusted the information provided by their physicians, compared to 36% in Finland. In Finland, 16% relied on information from HCWs other than doctors, while the European average was 9% [23]. In the Finnish Science Barometer 2022, 77% of Finns believed that anti-vaccination attitude was due to misinformation and prejudice [24]. In 2021, Finland had a relatively high vaccine coverage of over 18 years old; for example, the *SARS-CoV-2* vaccine coverage was around 89%, compared to the European average of 83% [25].

Research on vaccine acceptance among Finns has mainly focused on describing vaccine acceptance of specific vaccines or groups of people, and vaccine confidence. In order to better understand vaccine acceptance among Finnish adults, we multidimensionally described and explained barriers to vaccine acceptance in the general population aged 18–79 in mainland Finland.

## Methods

We conducted a secondary analysis of data from the Fimea Medicines Barometer 2021 cross-sectional population survey, which was prepared in cooperation with the Finnish Institute for Health and Welfare, the University of Turku, the University of Eastern Finland, and the Social Insurance Institution of Finland to describe the Finns’ perceptions of medicines, including vaccines. The Medicines Barometer is a biennial population survey conducted by Fimea on topical issues in pharmaceutical care. The aim of the survey is to describe and explain the experiences and perceptions of Finns aged 18–79 about medicines. The questionnaire has been piloted and modified based on feedback [26]. The online survey was sent to 10105 members of Taloustutkimus Oy’s closed Internet panel (recruited around 40000 people via multichannel) [27], selected by stratified random sampling based on age, gender, education level, and area of residence. The target was 2000 responses. The survey was carried out in the Finnish language between 23 and 30 September 2021 [26].

### Response rate and representativeness

The response rate to the original survey was 20.6% (*n* = 2081/10105 invited). Women were slightly over-represented compared to the general population (52% versus 50%). In terms of age, education level, and residential area, young people, those with low or medium levels of education, and those living in rural or suburban areas were underrepresented. Older people, those with higher education, and those living in cities were over-represented [26]. The number of responses included in the analysis ranged from 87% to 97.7%, depending on the number of missing values.

### Variables selected

We selected the following statements from the ‘Vaccines’ module: ‘Vaccination is a good way to protect against disease’, ‘Vaccines are safe’, ‘I have enough information about VPDs’, ‘I have enough information about vaccines’, ‘Everyone should get vaccinated according to the national vaccination schedule’, ‘Vaccination contradicts with my way of thinking’, ‘Anti-vaccination is a big problem in Finland’, and ‘The media deals with vaccination and vaccine-related issues responsibly’. The respondents expressed agreement or disagreement with the statements using a five-point Likert scale, with responses ranging from 1 (completely agree) to 4 (completely disagree); additionally, 5 (can’t say) was an option. We also used relevant socio-demographic variables, considering the Finnish context and the possibilities offered by the data (Table S1 in the Supplementary Material).

### Data management

We combined the response options ‘completely agree’ and ‘somewhat agree’ and did a similar process to disagreements, respectively. ‘Can’t say’ was the fifth non-neutral response option, so we considered them missing. For quantitative statistical analysis, we also considered gender ‘other’ as a missing value due to the small number of respondents (*n* = 20) to protect respondents’ anonymity in accordance with Finnish data protection legislation. For consistency of interpretation, the original statement ‘Vaccination contradicts with my way of thinking’ has been reversed in the same direction as the other statements (Table S1 in the Supplementary Material), except for factor analysis. The total number of respondents for each statement varies due to missing values.

### Statistical analysis

We conducted a descriptive analysis (*n*, %) and univariate comparison (chi-square test) of all statements (Tables S2 and S3 in the Supplementary Material). We conducted a single variable analysis adjusted for age (18–39, 40–59, 60–79), gender (woman; man), and residential area (Helsinki–Uusimaa, South Finland, North and East Finland, West Finland) for all variables of interest, using logistic regressions (Tables S4 and S5 in the Supplementary Material). Then, we included all variables under *p* < 0.25 in a multivariable model. We also conducted an exploratory factor analysis (EFA) to find latent patterns in the variables, which allowed us to identify similarities between the statements. We used R version 4.2.2 [28].

## Results

### Confidence in vaccines

Overall, 3% (63/2034) of respondents disagreed that ‘Vaccination is a good way to protect against disease’, reflecting trust in vaccine efficacy. Compared to older respondents, individuals aged 18–39 years and 40–59 years were more than twice as likely to disagree (OR = 2.4 (1.2–5.1) and OR = 2.2 (1.2–4.4), respectively, *p* = 0.014). Compared to participants with tertiary education, respondents with upper secondary education were more than twice as likely to disagree (OR = 2.1 (1.2–3.7), *p* = 0.005). Men were more likely than women to disagree (OR = 1.7 (1.0–2.9), *p* = 0.032). Finally, respondents with self-perceived poor or fairly poor health status were almost three times more likely to disagree with the statement (OR = 2.9 (1.2–6.3), *p* = 0.042) compared to participants with good or fairly good self-perceived health status (Table 1).

**Table 1.** Multivariable analysis: ‘Confidence in vaccines’ and ‘Access to information on vaccines and VPDs’

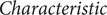	‘Vaccination is a good way to protect against disease’		‘Vaccine are safe’		‘I have enough information about VPDs’		‘I have enough information about vaccines’	
	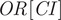	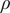	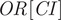	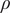	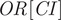	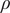	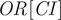	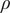
Age group								
60–79	Reference		Reference		Reference		Reference	
40–59	2.2 [1.2–4.4]	0.014*	3.2 [2.0–5.1]	<0.001***	1.6 [1.1–2.2]	<0.001***	1.4 [0.9–2.0]	<0.001***
18–39	2.4 [1.2–5.1]		3.1 [1.9–5.2]		2.8 [1.8–4.7]		2.0 [1.3–3.0]	
Gender								
Woman	Reference		Reference		Reference		Reference	
Man	1.7 [1.0–2.9]	0.032*	1.2 [0.8–1.7]	0.295	1.3 [1.0–1.8]	0.007**	1.0 [0.8–1.3]	0.555
Residential area								
Helsinki–Uusimaa	Reference		Reference		Reference		Reference	
South Finland	0.9 [0.4–1.7]	0.784	1.1 [0.7–1.8]	0.211	1.3 [0.9–2.0]	0.551	1.1 [0.8–1.5]	0.301
North and East Finland	0.7 [0.3–1.5]		0.9 [0.5–1.5]		1.3 [0.9–1.9]		1.1 [0.8–1.5]	
West Finland	0.7 [0.3–1.3]		1.4 [0.9–2.2]		1.2 [0.9–1.7]		1.3 [1.0–1.8]	
Marital status								
In relationship	Reference		Reference		Reference		Reference	
Relationship–free	1.5 [0.8–2.7]	0.088	1.1 [0.7–1.6]	0.095	1.3 [0.9–1.8]	0.001**	1.1 [0.8–1.4]	0.021*
Highest education								
Tertiary education	Reference		Reference		Reference		Reference	
Upper secondary education	2.1 [1.2–3.7]	0.005**	2.0 [1.3–2.9]	<0.001***	1.0 [0.6–1.6]	0.003**	1.5 [1.1–1.9]	<0.001***
First–degree education	0.6 [0.1–2.0]		1.4 [0.7–2.9]		1.4 [0.8–2.3]		1.5 [0.9–2.3]	
Household net income per month								
More than 5000 €	Reference		Reference		Reference		Reference	
2001–5000 €	0.5 [0.3–1.3]	0.140	1.3 [0.7–2.8]	0.022*	1.2 [0.8–1.9]	0.158	1.0 [0.7–1.5]	0.117
Up to 2000 €	0.7 [0.3–2.0]		1.7 [0.8–4.0]		1.0 [0.6–1.8]		1.4 [0.9–2.3]	
Don’t want to say or don’t know	1.2 [0.5–3.2]		2.6 [1.2–5.6]		0.9 [0.6–1.4]		1.3 [0.8–2.0]	
Health status								
Good or fairly good health	Reference		Reference		Reference		Reference	
Moderate health	1.6 [0.9–2.9]	0.042*	1.7 [1.1–2.4]	0.015*	1.0 [0.5–1.6]	0.001**	1.8 [1.4–2.3]	<0.001***
Poor or fairly poor health	2.9 [1.2–6.3]		1.7 [0.9–3.1]		1.6 [0.9–2.6]		1.8 [1.1–2.9]	
Principal activity								
Retired					Reference		Reference	
Working					1.3 [0.7–2.2]	0.508	1.1 [0.8–1.7]	0.489
Student or other					1.4 [0.8–2.4]		0.9 [0.5–1.4]	
Unemployment					1.3 [0.7–2.3]		1.3 [0.7–2.2]	
Urbanization level								
Urban	Reference		Reference				Reference	
Suburban	1.3 [0.5–2.7]	0.112	1.3 [0.7–2.1]	0.065			1.0 [0.7–1.4]	0.093
Rural	2.2 [1.0–4.4]		1.8 [1.1–2.9]				1.4 [1–0–2.0]	
Education in health care								
No			Reference		Reference		Reference	
Yes			0.7 [0.4–1.2]	0.170	2.1 [1.3–3.7]	0.002**	0.7 [0.4–0.9]	0.019*
Using the Internet to find health information								
No	Reference		Reference		Reference		Reference	
Yes	0.6 [0.3–1.2]	0.152	0.8 [0.5–1.4]	0.391	1.4 [0.9–2.0]	0.101	0.8 [0.6–1.1]	0.166
Social Insurance Institution’s special reimbursement for medicines								
No								
Yes								
*n*	2034		1990		1950		1931	
*Nagelkerke R^2^*	0.09		0.10		{0.09}		0.07	

***p < 0.001, **p < 0.01, *p < 0.05.

As far as vaccine safety is concerned, 7% (147/1990) of respondents disagreed with the statement that ‘Vaccines are safe’. We found similar associations with the statement on vaccination being a good way to protect against disease: younger participants were more likely to disagree that vaccines are safe, compared to participants aged 60–79 years (OR = 3.1 (1.9–5.2) for 18–39 years and OR = 3.2 (2.0–5.1) for 40–59 years, *p* < 0.001). Respondents with upper secondary education were twice more likely to disagree than respondents with tertiary education (OR = 2.0 (1.3–2.9), *p* < 0.001). To a lesser extent, self-perceived moderate health status increased the likelihood of disagreement with the statement (OR = 1.7 (1.1–2.4), *p* = 0.015). Correspondingly, in terms of net monthly household income, those unwilling or unable to say their household net income levels were most likely to disagree with the statement (OR = 2.6 (1.2–5.6), *p* = 0.022) compared to those household net income levels over 5000€ (Table 1).

### Access to information on vaccines and VPDs

A total of 14% (268/1950) of respondents disagreed that they had enough information about VPDs (statement: ‘I have enough information about VPDs’). Respondents aged 18–39 years (OR = 2.8 (1.8–4.7)) were three times more likely to disagree, while respondents aged 40–59 years were less than double (OR = 1.6 (1.1–2.2), *p* < 0.001) compared to respondents aged 60–79 years. Being a man compared to being a woman (OR = 1.3 (1.0–1.8), *p* = 0.007) and education in health care (OR = 2.1 (1.3–3.7), *p* = 0.002) were also significantly associated with the likelihood of disagreement (Table 1).

Overall, 20% (388/1931) of respondents disagreed that they had enough information about vaccines (statement: ‘I have enough information about vaccines’). Respondents aged 18–39 years stood out from the age groups in terms of likelihood to disagree (OR = 2.0 (1.3–3.0), *p* < 0.001). Upper secondary education compared to tertiary education (OR = 1.5 (1.1–1.9), *p* < 0.001), as well as poor or fairly poor self-perceived health (OR = 1.8 (1.1–2.9)) and moderate self-perceived health compared to good or fairly good self-perceived health (OR = 1.8 (1.4–2.3), *p* < 0.001), increased the likelihood of disagreement. Conversely, healthcare education reduced the likelihood of disagreement by 30% (OR = 0.7 (0.4–0.9), *p* = 0.019) (Table 1).

### Debate on vaccination issues

Overall, 5% (104/2020) of respondents disagreed that ‘Everyone should get vaccinated according to the national vaccination schedule’. Younger respondents were almost three times more likely to disagree compared to those aged 60–79 (OR = 2.6 (1.2–5.8) for those aged 18–39 and OR = 2.9 (1.4–6.1) for those aged 40–59, *p* < 0.001). Men were twice more likely to disagree compared to women (OR = 2.0 (1.3–3.1), *p* < 0.001). The poorer the self-perceived health of the respondent, the more likely they were to disagree (OR = 2.1 (1.0–4.2) for poor or fairly poor self-perceived health and OR = 1.7 (1.1–2.7) for moderate self-perceived health, *p* = 0.022). Compared to retired respondents, the likelihood to disagree increased for unemployment (OR = 2.9 (1.2–7.2)) and for studying or other (OR = 3.0 (1.4–6.9), *p* = 0.016). In contrast, healthcare education reduced the likelihood of disagreeing by 50% (OR = 0.5 (0.2–1.0), *p* = 0.039) (Table 2).

**Table 2.** Multivariable analysis: ‘Debate on vaccination issues’

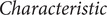	‘Everyone should get vaccinated according to the national vaccination schedule’		‘Vaccination does not contradict with my way of thinking’		‘Anti-vaccination is a big problem in Finland’		‘The media deals with vaccination and vaccine-related issues responsibly’	
	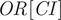	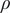	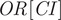	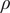	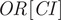	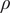	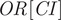	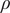
Age group								
60–79	Reference		Reference		Reference		Reference	
40–59	2.9 [1.4–6.1]	<0.001***	3.5 [1.8–6.9]	0.017*	1.7 [1.2–2.5]	<0.001***	1.8 [1.4–2.4]	<0.001***
18–39	2.6 [1.2–5.8]		3.7 [1.8–7.8]		2.0 [1.4–2.9]		2.3 [1.8–3.1]	
Gender								
Woman	Reference		Reference		Reference		Reference	
Man	2.0 [1.3–3.1]	<0.001***	0.9 [0.6–1.3]	0.801	1.2 [1.0–1.5]	0.101	1.3 [1.1–1.6]	0.011*
Residential area								
Helsinki–Uusimaa	Reference		Reference		Reference		Reference	
South Finland	1.9 [1.1–3.3]	0.066	1.1 [0.6–1.8]	0.784	1.1 [0.8–1.4]	0.973	1.4 [1.1–1.9]	0.014*
North and East Finland	1.2 [0.7–2.3]		1.1 [0.7–1.9]		1.0 [0.7–1.4]		1.3 [1.0–1.8]	
West Finland	1.6 [0.9–2.7]		1.0 [0.6–1.7]		1.1 [0.8–1.4]		1.2 [0.9–1.6]	
Marital status								
In relationship	Reference							
Relationship–free	1.1 [0.7–1.9]	0.104						
Highest education								
Tertiary education	Reference		Reference				Reference	
Upper secondary education	1.4 [0.9–2.3]	0.036*	1.3 [0.8–1.9]	0.049*			1.4 [1.1–1.8]	<0.001***
First–degree education	0.8 [0.2–1.9]		1.5 [0.7–3.0]				1.7 [1.1–2.5]	
Household net income per month								
More than 5000 €	Reference		Reference				Reference	
2001–5000 €	1.3 [0.6–2.9]	0.113	1.2 [0.7–2.5]	0.431			1.1 [0.8–1.5]	0.051
Up to 2000 €	1.2 [0.5–3.2]		1.1 [0.5–2.5]				0.8 [0.6–1.2]	
Don’t want to say or don’t know	2.2 [0.9–5.4]		1.7 [0.8–3.7]				1.4 [1.0–2.1]	
Health status								
Good or fairly good health	Reference				Reference		Reference	
Moderate health	1.7 [1.1–2.7]	0.022*			0.8 [0.6–1.1]	0.079	1.5 [1.1–1.8]	0.005**
Poor or fairly poor health	2.1 [1.0–4.2]				0.8 [0.5–1.3]		1.5 [0.9–2.3]	
Principal activity								
Retired	Reference		Reference		Reference			
Working	1.5 [0.7–3.4]	0.016*	0.4 [0.2–0.8]	0.072	1.6 [1.1–2.3]	0.002**		
Student or other	3.0 [1.4–6.9]		0.5 [0.2–1.2]		2.1 [1.4–3.2]			
Unemployment	2.9 [1.2–7.2]		0.6 [0.3–1.5]		2.0 [1.2–3.4]			
Urbanization level								
Urban			Reference				Reference	
Suburban			1.1 [0.6–1.9]	0.203			1.1 [0.8–1.5]	0.095
Rural			1.6 [1.0–2.7]				1.4 [1.0–2.0]	
Education in health care								
No	Reference				Reference			
Yes	0.5 [0.2–1.0]	0.039*			0.7 [0.5–1.0]	0.034*		
Using the Internet to find health information								
No	Reference		Reference					
Yes	0.6 [0.4–1.1]	0.118	0.6 [0.4–1.0]	0.042*				
Social Insurance Institution’s special reimbursement for medicines								
No					Reference			
Yes					0.8 [0.6–1.0]	0.056		
*n* *Nagelkerke R^2^*	2020 0.13		1996 0.05		1906 0.09		1810 0.06	

***p < 0.001, **p < 0.01, *p < 0.05.

Regarding the statement ‘Vaccination does not contradict with my way of thinking’, 6% (118/1996) disagreed. Younger respondents disagreed almost four times more likely than 60–79-year-old respondents (OR = 3.7 (1.8–7.8) for 18–39 years old and OR = 3.5 (1.8–6.9) for 40–59 years old, *p* = 0.017). On the contrary, using the Internet to find health information reduced the likelihood of disagreement by 40% (OR = 0.6 (0.4–1.0), *p* = 0.042) (Table 2).

Overall, 27% (517/1906) disagreed that ‘Anti-vaccination is a big problem in Finland’. The likelihood of disagreeing increased about twice (OR = 2.0 (1.4–2.9) for those aged 18–39 and OR = 1.7 (1.2–2.5) for those aged 40–59, *p* < 0.001) compared to those aged 60–79. The probability of disagreement increased in all other main activity categories compared to retired respondents (OR = 2.0 (1.2–3.4) for unemployed, OR = 2.1 (1.4–3.2) for student or other, and OR = 1.6 (1.1–2.3) for working, *p* = 0.002). Healthcare education, on the other hand, reduced the likelihood of disagreeing by 30% (OR = 0.7 (0.5–1.0), *p* = 0.034) (Table 2).

In total, 29% of respondents (532/1810) disagreed that ‘The media deals with vaccination and vaccine-related issues responsibly’. Being either young or middle-aged increased the likelihood to disagree about twice compared to 60–79 years old (OR = 2.3 (1.8–3.1) for those aged 18–39 and OR = 1.8 (1.4–2.4) for those aged 40–59, *p* < 0.001). Lower level of education increased the likelihood of disagreeing compared to tertiary education (OR = 1.7 (1.1–2.5) for first-degree education and OR = 1.4 (1.1–1.8) for upper secondary education, *p* < 0.001), respectively. Moderate self-perceived health compared to good or fairly good self-perceived health increased the likelihood to disagree (OR = 1.5 (1.1–1.8), *p* = 0.005). Men were 30% more likely to disagree than women (OR = 1.3 (1.1–1.6), *p* = 0.011). Similarly, people living outside the Finnish metropolitan area (Helsinki–Uusimaa) were 30–40% more likely to disagree with the statement than people living in Helsinki–Uusimaa (OR = 1.3 (1.0–1.8) for North and East Finland and OR 1.4 (1.1–1.9) for South Finland, *p* = 0.014) (Table 2).

### Exploratory factor analysis

**Figure 1.**
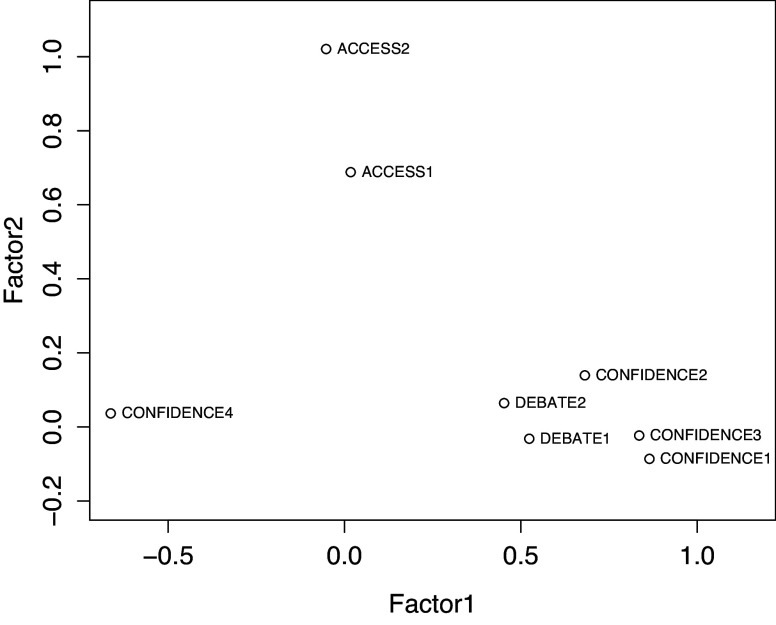
Distribution of statements into factors in a two-factor dimension with oblique rotation.

We conducted an EFA to find the dimensions within the variables. The two-factor model did not fit the model, so we added a third factor. The three-factor model showed the best fit ( 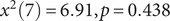 ), explaining 50% of the total variation in the data. We identified factors related to ‘Confidence and attitudes towards vaccines’, ‘Access to information on vaccines and VPDs’, and ‘Debate on vaccination issues’. Factors related to ‘Access to information on vaccines and VPDs’ and ‘Debate on vaccination issues’ showed a strong correlation, *r* = 0.80. The factor ‘Confidence and attitudes towards vaccines’ was moderately correlated with the factor ‘Debate on vaccination issues’, *r* = 0.49, as was the factor ‘Access to information on vaccines and VPDs’, *r* = 0.43. Communality was high for the statements ‘Vaccination is a good way to protect against diseases’, *r* = 0.74, and ‘I have enough information about vaccines’, *r* = 1.00 (Tables 3 and 4 and Figure 1).

**Table 3.** Exploratory factor analysis factor loadings using Promax rotation: we generated three factors of vaccine acceptance from the loadings of the variables that reflected the underlying similarity of the responses

Estimated classification of statements	Factor loadings (cut-off 0.30)			Communality	Statement codes
	Factor 1	Factor 2	Factor 3		
Confidence in vaccines					
Vaccination is a good way to protect against disease	**0.86**			0.74	CONFIDENCE1
Vaccines are safe	**0.37**		0.35	0.28	CONFIDENCE2
Access to information on vaccines and VPDs					
I have enough information about VPDs		**0.69**		0.47	ACCESS1
I have enough information about vaccines		**1.00**		1.00	ACCESS2
Debate on vaccination issues					
Everyone should get vaccinated according to the national vaccination schedule	**0.50**		0.41	0.43	CONFIDENCE3
Vaccination contradicts with my way of thinking	**−0.64**			0.41	CONFIDENCE4
Anti–vaccination is a big problem in Finland			**0.67**	0.46	DEBATE1
The media deals with vaccination and vaccine–related issues responsibly			**0.33**	0.14	DEBATE2
Eigenvalue	1.57	1.56	0.85		
Explanation rate	0.20	0.19	0.11		

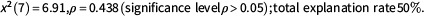

Highest values in bold.

**Table 4.** Factor correlations

	Factor 1	Factor 2	Factor 3
Factor 1		0.43	0.49
Factor 2			0.80
Factor 3

## Discussion

Our main findings showed that barriers to vaccine acceptance among the Finnish adult population included younger age, lower education, poorer health, and male gender. In addition to lower education, we found that other socioeconomic backgrounds associated with vulnerability, such as unemployment and studying, were significant barriers to vaccine acceptance. We found that vulnerable status is a major barrier to vaccine acceptance, either interactively among multiple socio-demographic variables or as a single factor.

### Socio-demographic barriers to vaccine acceptance

We found that low and medium levels of education are barriers to vaccine acceptance, which is in line with a major European study showing that low levels of education are associated with social vulnerability in many Finnish regions and that increased spending on education and improved political stability would reduce vulnerability [14]. Our results are also in line with other previous studies from Europe, where vaccine hesitancy was associated with young age, male gender, unmarried, unemployment, and low income, in addition to lower education. Higher education is also strongly associated with vaccine hesitancy in previous studies, often for ideological reasons. These include institutional distrust, linked to political extremism, and information-seeking behaviour, as those who use social networks to seek health-related information are often more hesitant. Vaccine hesitancy among higher educated people is also often linked to hesitation towards science and scientists, or religious beliefs and misinformation about health [6, 17, 20, 29]. In recent years, attitudes towards science, researchers, and health authorities have become polarized, similar to those towards vaccines. Even though Finnish adults strongly favour the use of science to support policymaking, such as vaccination policies, Finnish surveys conducted in 2020–2021 show that 22% of Finns were suspicious of the vaccine research institutions, such as the Finnish Institute for Health and Welfare and the Ministry of Social Affairs and Health, respectively, and 26% doubted the reporting of the news media and the Finnish government on the *coronavirus* pandemic [6], which is in line with our findings.

Vaccines under the NIP are free of charge in Finland [30], which supports our finding that the household monthly net income was not a significant factor for any of the statements in the multivariable analysis. However, previous studies have found that low household net income was often a barrier to vaccine uptake [15, 31], which is consistent with our single variable results, where monthly household net income up to 2000€ and 2001–5000€ significantly increased disagreement with statements on vaccine safety, having sufficient information about vaccines and VPDs compared to monthly household net income above 5000€. It is possible that the net income level of a household has an impact on vaccine acceptance in terms of access to information and the opportunity to be educated. Several serious diseases and medical histories have been found to be associated with lower vaccination uptake for *SARS-CoV-2* vaccine, in line with our findings that poorer health is associated with barriers to vaccine acceptance [14–16]. People with poorer medical conditions may be more hesitant due to increased worry about possible adverse reactions to vaccines [5, 32, 33]. Living outside Finland’s metropolitan areas has been found to increase barriers to vaccine acceptance [5, 34], which is in line with our findings on the media’s responsibility. Correspondingly, living in densely populated cities has been found to be associated with lower vaccine hesitancy compared to rural and suburban areas [8, 14, 29, 31]. We found that living outside the city was not significant in the multivariable analysis, but in the single variable analysis, living in a rural area increased disagreement with the statements about the media’s responsibility and vaccine safety. People may face practical challenges due to the distance between health service facilities and the area where they live.

### Barriers to vaccine acceptance related to receiving and seeking information

Our results indicate that healthcare education reduced the likelihood of barriers to vaccine acceptance for statements about sufficient vaccine information, anti-vaccination, and media responsibility on vaccine issues, while it increased barriers to acceptance on the statement about sufficient information on VPDs, which may reflect discrepancies in access to and understanding of information. A similar finding has been made in recent studies of unvaccinated HCWs in Finland, which found that negative vaccine recommendations from social networks reduced uptake of the *SARS-CoV-2* vaccine, compared to those who trusted health professionals [35], while people who trusted the Internet were more likely to be hesitant about the vaccine than those who trusted the media [19, 29]. Correspondingly, our study showed that using the Internet to find health information reduced disagreement with the statement ‘Vaccination does not contradict with my way of thinking’, which may be explained by the large number of official health information websites in Finland that are considered reliable [36]. On the contrary, the use of the Internet to find health information increased disagreement with the statement ‘I have enough information about VPDs’ in the single variable analysis, which may reflect an increase in misinformation and disinformation competing with official websites [19, 31, 36].

### Exploratory factor analysis

The EFA strongly supported the original assumption of a factor distribution of statements. Factors particularly explained the statements on access to information on vaccines and confidence in the efficacy of vaccines. Similarly, the factor on access to information on vaccines and VPDs correlated strongly with the factor on debate on vaccination issues, indicating the polarization of Finnish people’s vaccine acceptance with other social issues, in line with other European countries [6, 17, 20, 29].

### Strengths and limitations

Our study had several limitations. First, the sample used in this study has been selected from a closed community of about 40000 Finns, who were part of Taloustutkimus Oy’s Internet panel. There was an uneven distribution of respondents´ backgrounds; hence, the results are not fully generalizable to the population. Second, self-reported socio-demographic background may be reported for social approval. On the other hand, for example, in the case of household income, a significant proportion of respondents answered honestly that they did not want to disclose their income level. Third, the results cannot be used to identify actual vaccination behaviour, which also depends on a number of other factors that were not asked in the survey. Fourth, we used only quantitative methods to study the secondary data. Finally, the response rate was low (20.6%) and the exclusion of ‘can’t say’ responses may affect the interpretation. The number of responses excluded from the analysis by statement was quite low (2.3–8.4%), with the exception of the statement on media responsibility in dealing with vaccine issues (13%). However, the ‘no opinion’ (‘can’t say’) option has not been found to have a significant impact on the validity of the results [37].

One of the strengths of our study is that the original survey has been piloted and the vaccine questionnaire has already been administered twice [26]. Furthermore, unlike most previous studies on vaccine acceptance, we measured barriers to vaccine acceptance from a general perspective, without restriction to a specific group of people or vaccine. Thus, the general context of vaccine acceptance becomes more apparent, although the *coronavirus* pandemic of recent years and public debate are likely to influence the orientation of responses. Finally, our study was conducted in collaboration with a multidisciplinary working group, thus considering expert perspectives from different disciplines, which is in line with the recommended approach to study vaccine acceptance and hesitancy [38].

## Conclusions

We found that the majority of Finnish adult people are confident about vaccines and vaccinations, but there is clear hesitation in certain groups, especially with regard to vaccine information and trust in information providers. However, the results should be used with caution. Vaccine acceptance is constantly changing. Similarly, perceptions are abstract patterns of thinking that are influenced by social interaction and can change. The survey must be repeated at regular intervals to assess the current situation. The overlap between the latent dimensions of acceptance revealed that the Finnish barriers to vaccine acceptance are multidimensional entities based on knowledge and communication, in which social interaction plays an important role. Finnish adults’ perceptions of vaccines seem to have become polarized in the societal debate on vaccines; thus, gaps in vaccine communication and knowledge appear to be underlying barriers to vaccine acceptance among Finns. Further research could aim to develop vaccine communication between citizens and policymakers to increase vaccine uptake. In the future, it will be useful to identify the ideological patterns of thought that underlie vaccination behaviour, in relation to both vaccine acceptance and the wider trust of individuals and groups in authorities, science, and institutions, in order to gain perspectives to make vaccine information more understandable.

## Supporting information

Lasander et al. supplementary material Lasander et al. supplementary material

## Data Availability

The data used are available for research purposes by separate application to Fimea.

## Supplementary material

For supplementary material accompanying this paper visit http://doi.org/10.1017/S0950268824000463. click here to view supplementary material
