# Barriers to vaccine acceptance in the adult population of mainland Finland, 2021 – ERRATUM

**DOI:** 10.1017/S0950268824000621

**Published:** 2024-05-23

**Authors:** Mervi Lasander, Kimmo Elo, Katja Joronen, Timothée Dub

When this article was published in Epidemiology & Infection it contained errors in the display of Tables 1 and 2, where lines that should’ve been included were missing. This article has been updated to correct this.

The publisher apologises for this error.
