# Supplementary material for: Barriers to vaccine acceptance in the adult population of mainland Finland, 2021
Source: Epidemiol Infect. 2024 Mar 15;152:e54. doi: 10.1017/S0950268824000463 (PMC11022264; doi:10.1017/S0950268824000463)
Supplement: Lasander et al. supplementary material [file S0950268824000463sup001.pdf]

Epidemiology & Infection

Barriers to vaccine acceptance in adult population of mainland Finland, 2021

Lasander Mervi, Elo Kimmo, Joronen Katja, Dub Timothée

Supplementary material

Table S1 Data collected from Fimea´s Medicine Barometer 2021: Background information and statements

ORIGINAL STATEMENT	ORIGINAL ANSWER OPTIONS	MODIFIED STATEMENT	MODIFIED ANSWER CATEGORIES
Everyone should get vaccinated according to the national vaccination schedule	1 I completely agree<br>2 I somewhat agree<br>3 I somewhat disagree<br>4 I completely disagree<br>5 I can´t say		0 Agree (1 and 2)<br>1 Disagree (3 and 4)
Vaccines are safe	1 I completely agree<br>2 I somewhat agree<br>3 I somewhat disagree<br>4 I completely disagree<br>5 I can´t say		0 Agree (1 and 2)<br>1 Disagree (3 and 4)
Vaccination is a good way to protect against disease	1 I completely agree<br>2 I somewhat agree<br>3 I somewhat disagree<br>4 I completely disagree<br>5 I can´t say		0 Agree (1 and 2)<br>1 Disagree (3 and 4)
I have enough information about VPDs	1 I completely agree<br>2 I somewhat agree<br>3 I somewhat disagree<br>4 I completely disagree<br>5 I can´t say		0 Agree (1 and 2)<br>1 Disagree (3 and 4)
I have enough information about vaccines	1 I completely agree<br>2 I somewhat agree		0 Agree (1 and 2)<br>1 Disagree (3 and 4)

	3 I somewhat disagree<br>4 I completely disagree<br>5 I can´t say		
Vaccination contradicts with my way of thinking	1 I completely agree<br>2 I somewhat agree<br>3 I somewhat disagree<br>4 I completely disagree<br>5 I can´t say	Vaccination does not contradict with my way of thinking	0 Agree (3 and 4)<br>1 Disagree (1 and 2)
Anti-vaccination is a big problem in Finland	1 I completely agree<br>2 I somewhat agree<br>3 I somewhat disagree<br>4 I completely disagree<br>5 I can´t say		0 Agree (1 and 2)<br>1 Disagree (3 and 4)
The media deals with vaccination and vaccine-related issues responsibly	1 I completely agree<br>2 I somewhat agree<br>3 I somewhat disagree<br>4 I completely disagree<br>5 I can´t say		0 Agree (1 and 2)<br>1 Disagree (3 and 4)
What is your gender?	1 Women<br>2 Men<br>3 Other		1 Women<br>2 Men
Your year of birth?			1 18-39<br>2 40-59<br>3 60-79
What is your marital status?	1 Married, cohabitation or registered partnership<br>2 Unmarried<br>3 Divorced or separated<br>3 Widow		1 In relationship (1)<br>2 Relationship-free (2 and 3)
What do you do full time?	1 I work full time<br>2 I work part time<br>3 I´m partly at work, partly retired<br>4 I´m unemployed or laid off<br>5 I´m retired		1 Retired (5)<br>2 Working (1, 2 and 3)<br>3 Unemployed (4)<br>4 Student or other activity (6, 7, 8 and 9)

	6 I´m on maternity, paternity, parental or care leave<br>7 I´m studying<br>8 I´m on long-term (>6 months) sick leave<br>9 Something else	
What is your highest level of education?	1 Primary school<br>2 Upper secondary school<br>3 Vocational school<br>4 University of applied sciences<br>5 University or other institute for higher education<br>6 Other	1 First degree education (1 and 6)<br>2 Upper secondary education (2 and 3)<br>3 Tertiary education (4 and 5)
Do you have any education in health care?	1 No<br>2 Yes	
What is your current residential area?	1 The center or suburb of the Helsinki Metropolitan Area (Helsinki, Espoo, Vantaa, Kauniainen)<br>2 Some other city center or suburb<br>3 Municipal center, church village or agglomeration<br>4 Rural areas	1 Urban (1 and 2)<br>2 Suburban (3)<br>3 Rural (4)
What is the total disposable monthly income (after-tax income and benefits) of the members of your household?	1 At the most 1000 €<br>2 1001–2000 €<br>3 2001–3000 €<br>4 3001–4000 €<br>5 4001–5000 €<br>6 5001–8000 €<br>7 Over 8000 €<br>8 I can´t say<br>9 I don´t want to answer	1 Up to 2000 €<br>2 2001-5000 €<br>3 More than 5000 €<br>4 Don't want to say or don't know
Do you use the internet to search for health information?	1 No<br>2 Yes	

What is your current health status?	1 Good<br>2 Fairly good<br>3 Moderate<br>4 Fairly poor<br>5 Poor	1 Good or fairly good<br>2 Moderate<br>3 Poor or fairly poor
Do you have any disease that entitles you to special reimbursement for medicines provided by the Finnish Social Insurance Institute?	1 No<br>2 Yes	

Table S2 Univariate comparison: "Confidence in vaccines" and "Access to information on vaccines and VPDs"

	**"Vaccination is a good way to protect against disease"**			**"Vaccine are safe"**			**"I have enough information about VPDs"**			**"I have enough information about vaccines"**		
Characteristic	*Agree n (%)*	*Dis-agree n (%)*	*ρ*	*Agree n (%)*	*Dis-agree n (%)*	*ρ*	*Agree n (%)*	*Dis-agree n (%)*	*ρ*	*Agree n (%)*	*Dis-agree n (%)*	*ρ*
Age group												
60-79	821 (98)	15 (2)		784 (96)	31 (4)		707 (90)	74 (10)		640 (84)	123 (16)	
40-59	688 (96)	29 (4)	0.021*	632 (90)	71 (10)	<0.001***	595 (86)	96 (14)	<0.001***	552 (79)	143 (21)	<0.001***
18-39	482 (96)	19 (4)		445 (91)	45 (9)		395 (79)	102 (21)		368 (75)	123 (25)	
Gender												
Woman	1046 (98)	26 (2)	0.086	970 (93)	74 (7)	0.653	906 (88)	125 (12)	0.033*	817 (80)	203 (20)	0.869
Man	925 (96)	37 (4)		873 (92)	73 (8)		776 (84)	143 (16)		726 (80)	185 (20)	
Residential area												
Helsinki-Uusimaa	747 (97)	26 (3)		707 (94)	48 (6)		643 (86)	101 (14)		600 (82)	133 (18)	
South Finland	400 (97)	14 (3)	0.797	375 (92)	31 (8)	0.253	341 (87)	52 (13)	0.549	310 (79)	80 (21)	0.314
North and East Finland	367 (97)	11 (3)		347 (93)	24 (7)		322 (87)	46 (13)		293 (80)	72 (20)	
West Finland	477 (97)	12 (3)		432 (91)	44 (9)		391 (84)	73 (16)		357 (77)	104 (23)	
Marital status												
In relationship	1256 (97)	32 (3)	0.064	1184 (94)	81 (6)	0.049*	1098 (89)	140 (11)	<0.001***	1008 (82)	222 (18)	0.007**
Relationship-free	735 (96)	31 (4)		677 (91)	66 (9)		599 (82)	132 (18)		522 (77)	167 (23)	
Highest education												
Tertiary education	1015 (98)	21 (2)	0.003**	972 (95)	48 (5)	<0.001***	893 (89)	112 (11)	0.002**	837 (84)	160 (16)	<0.001***
Upper secondary education	837 (95)	40 (5)		765 (90)	88 (10)		699 (83)	138 (17)		627 (76)	196 (24)	

First degree education	139 (99)	2 (1)		124 (92)	11 (8)		105 (83)	22 (17)		96 (74)	33 (26)	
Household net income per month												
More than 5000 €	279 (97)	9 (3)		272 (96)	11 (4)		249 (89)	32 (11)		238 (84)	44 (16)	
2001-5000 €	1045 (98)	23 (2)		985 (94)	64 (6)		905 (88)	118 (12)		837 (82)	183 (18)	
Up to 2000 €	395 (96)	17 (4)	0.051	361 (90)	41 (10)	<0.001***	317 (80)	79 (20)	<0.001***	281 (73)	105 (27)	<0.001***
Don't want to say or don't know	272 (95)	14 (5)		243 (89)	31 (11)		226 (84)	43 (16)		204 (78)	57 (22)	
Health status												
Good or fairly good health	1353 (97)	35 (3)		1280 (94)	83 (6)		1177 (88)	159 (12)		1103 (83)	224 (17)	
Moderate health	527 (96)	20 (4)	0.025*	477 (90)	51 (10)	0.008**	430 (82)	91 (18)	0.001**	377 (74)	134 (26)	<0.001***
Poor or fairly poor health	111 (93)	8 (7)		104 (89)	13 (11)		90 (80)	22 (20)		80 (72)	31 (28)	
Principal activity												
Retired	695 (98)	16 (2)		664 (95)	33 (5)		595 (89)	74 (11)		537 (82)	115 (18)	
Working	920 (97)	29 (3)	0.188	851 (92)	76 (8)	0.006**	796 (87)	117 (13)	<0.001***	737 (80)	180 (20)	0.010*
Student or other	266 (96)	12 (4)		248 (91)	25 (9)		220 (80)	54 (20)		210 (78)	60 (22)	
Unemployment	110 (95)	6 (5)		98 (88)	13 (12)		85 (76)	27 (24)		76 (69)	34 (31)	
Urbanization level												
Urban	1540 (97)	43 (3)		1449 (93)	100 (7)		1308 (86)	210 (14)		1211 (81)	286 (19)	
Suburban	214 (96)	8 (4)	0.179	197 (91)	19 (9)	0.013*	189 (87)	28 (13)	0.881	173 (80)	42 (20)	0.059
Rural	237 (95)	12 (5)		215 (88)	28 (12)		200 (85)	34 (15)		176 (74)	61 (26)	
Education in health care												
No	1732 (97)	57 (3)	0.534	1616 (92)	133 (8)	0.254	1455 (85)	253 (15)	0.001**	1340 (79)	352 (21)	0.021*
Yes	259 (98)	6 (2)		245 (95)	14 (5)		242 (93)	19 (7)		220 (86)	37 (14)	

Using internet to find health information												
No	271 (95)	13 (5)	0.160	249 (92)	22 (8)	0.677	218 (83)	44 (17)	0.160	203 (77)	59 (23)	0.302
Yes	1720 (97)	50 (3)		1612 (93)	125 (7)		1479 (87)	228 (13)		1357 (80)	330 (20)	
Social Insurance Institution´s special reimbursement for medicines												
No	1365 (97)	43 (3)	1.000	1279 (93)	101 (7)	1.000	1159 (86)	190 (14)	0.658	1078 (80)	267 (20)	0.908
Yes	626 (97)	20 (3)		582 (93)	46 (7)		538 (87)	82 (13)		482 (80)	122 (20)	

$*** \rho < 0.001$ $** \rho < 0.01$ $* \rho < 0.05$

Table S3 Univariate comparison: "Debate on vaccination issues"

	**"Everyone should get vaccinated according to the national vaccination schedule"**			**"Vaccination does not contradict with my way of thinking"**			**"Anti-vaccination is a big problem in Finland"**			**"The media deals with vaccination and vaccine-related issues responsibly"**		
Characteristic	*Agree n (%)*	*Dis−agree n (%)*	*ρ*	*Agree n (%)*	*Dis−agree n (%)*	*ρ*	*Agree n (%)*	*Dis−agree n (%)*	*ρ*	*Agree n (%)*	*Dis−agree n (%)*	*ρ*
Age group												
60-79	818 (98)	19 (2)		786 (96)	34 (4)		656 (84)	126 (16)		584 (77)	174 (23)	
40-59	657 (93)	49 (7)	<0.001***	656 (93)	51 (7)	0.021*	447 (68)	213 (32)	<0.001***	419 (67)	202 (33)	<0.001***
18-39	459 (92)	38 (8)		454 (93)	34 (7)		298 (62)	186 (38)		283 (63)	165 (37)	
Gender												

Woman	1026 (96)	40 (4)	0.004**	989 (94)	65 (6)	0.677	719 (74)	258 (26)	0.502	671 (73)	251 (27)	0.044*
Man	890 (93)	64 (7)		889 (94)	53 (6)		670 (72)	259 (28)		607 (68)	281 (32)	
Residential area												
Helsinki-Uusimaa	735 (96)	29 (4)	0.087	714 (95)	40 (5)	0.792	532 (73)	200 (27)	0.943	513 (74)	177 (26)	0.027*
South Finland	380 (93)	29 (7)		384 (94)	26 (6)		281 (74)	100 (26)		241 (67)	121 (33)	
North and East Finland	358 (95)	19 (5)		349 (93)	25 (7)		256 (73)	95 (27)		231 (67)	111 (33)	
West Finland	461 (94)	29 (6)		449 (94)	28 (6)		332 (72)	130 (28)		301 (69)	132 (31)	
Marital status												
In relationship	1227 (96)	56 (4)	0.036*	1194 (94)	69 (6)	0.320	886 (74)	310 (26)	0.102	819 (70)	345 (30)	1.000
Relationship-free	707 (93)	50 (7)		702 (93)	50 (7)		515 (70)	215 (30)		467 (70)	196 (30)	
Highest education												
Tertiary education	991 (96)	41 (4)	0.012*	977 (95)	49 (5)	0.076	706 (72)	268 (28)	0.166	685 (74)	243 (26)	0.004**
Upper secondary education	810 (93)	60 (7)		796 (93)	59 (7)		592 (72)	231 (28)		523 (67)	256 (33)	
First degree education	133 (96)	5 (4)		123 (92)	11 (8)		103 (80)	26 (20)		78 (65)	42 (35)	
Household net income per month												
More than 5000 €	277 (97)	9 (3)	0.023*	276 (96)	12 (4)	0.241	203 (74)	72 (26)	0.419	195 (74)	68 (26)	0.121
2001-5000 €	1016 (96)	47 (4)		995 (94)	59 (6)		740 (74)	263 (26)		677 (70)	284 (30)	
Up to 2000 €	376 (93)	29 (7)		372 (93)	26 (7)		279 (72)	109 (28)		259 (71)	104 (29)	
Don't want to say or don't know	265 (93)	21 (7)		253 (92)	22 (8)		179 (69)	81 (31)		155 (65)	85 (35)	
Health status												
Good or fairly good health	1322 (96)	59 (4)	0.007**	1290 (94)	77 (6)	0.406	924 (70)	387 (30)	0.005**	909 (73)	342 (27)	0.007**
Moderate health	505 (93)	35 (7)		501 (94)	32 (6)		389 (78)	112 (22)		313 (66)	164 (34)	

Poor or fairly poor health	107 (90)	12 (10)		105 (91)	10 (9)		88 (77)	26 (23)		64 (65)	35 (35)	
Principal activity												
Retired	697 (98)	17 (2)		661 (94)	39 (6)		561 (85)	96 (15)		500 (78)	142 (22)	
Working	890 (95)	48 (5)	<0.001***	879 (94)	51 (6)	0.277	601 (67)	289 (33)	<0.001***	559 (67)	279 (33)	<0.001***
Student or other	248 (90)	27 (10)		256 (93)	18 (7)		169 (63)	101 (37)		164 (66)	85 (34)	
Unemployment	99 (88)	14 (12)		100 (90)	11 (10)		70 (64)	39 (36)		63 (64)	35 (36)	
Urbanization level												
Urban	1494 (95)	76 (5)		1467 (95)	82 (5)		1082 (73)	403 (27)		1014 (72)	395 (28)	
Suburban	210 (95)	12 (5)	0.278	207 (94)	14 (6)	0.040*	153 (71)	61 (29)	0.907	135 (67)	65 (33)	0.015**
Rural	230 (93)	18 (7)		222 (91)	23 (9)		166 (73)	61 (27)		137 (63)	81 (37)	
Education in health care												
No	1678 (94)	99 (6)	0.067	1653 (94)	104 (6)	1.000	1210 (72)	467 (28)	0.153	1120 (70)	470 (30)	0.961
Yes	256 (97)	7 (3)		243 (94)	15 (6)		191 (77)	58 (23)		166 (70)	71 (30)	
Using internet to find health information												
No	264 (93)	19 (7)	0.273	256 (92)	23 (8)	0.099	192 (74)	69 (26)	0.806	177 (70)	75 (30)	1.000
Yes	1670 (95)	87 (5)		1640 (94)	96 (6)		1209 (73)	456 (27)		1109 (70)	466 (30)	
Social Insurance Institution´s special reimbursement for medicines				786 (96)	34 (4)	0.021*						
No	1315 (94)	79 (6)	0.193	656 (93)	51 (7)	0.658	912 (69)	404 (31)	<0.001***	869 (70)	379 (30)	0.324
Yes	619 (96)	27 (4)		454 (93)	34 (7)		489 (80)	121 (20)		417 (72)	162 (28)	

$*** \rho < 0.001$ $** \rho < 0.01$ $* \rho < 0.05$

Table S4 Single variable analysis adjusted for age, gender and region: “Confidence in vaccines” and “Access to information on vaccines and VPDs”

	**"Vaccination is a good way to protect against disease"**		**"Vaccine are safe"**		**"I have enough information about VPDs"**		**"I have enough information about vaccines"**	
Characteristic	$OR\ [CI]$	ρ	$OR\ [CI]$	ρ	$OR\ [CI]$	ρ	$OR\ [CI]$	ρ
Marital status								
In relationship	Reference		Reference		Reference		Reference	
Relationship-free	1.6 [0.9-2.6]	0.088	1.4 [1.0-1.9]	0.095	1.6 [1.2-2.0]	0.001**	1.3 [1.0-1.7]	0.021*
Highest education								
Tertiary education	Reference		Reference		Reference		Reference	
Upper secondary education	2.4 [1.4-4.1]	0.003**	2.4 [1.7-3.5]	<0.001***	1.2 [0.7-2.0]	<0.001***	1.6 [1.3-2.1]	<0.001***
First degree education	0.8 [0.1-2.8]		2.1 [1.0-4.1]		2.0 [1.2-3.2]		2.0 [1.3-3.0]	
Household net income per month								
More than 5000 €	Reference		Reference		Reference		Reference	
2001-5000 €	0.8 [0.4-1.9]		1.8 [1.0-3.7]		1.8 [1.3-2.5]		1.2 [0.8-1.7]	
Up to 2000 €	1.5 [0.7-3.7]	0.070	2.9 [1.5-6.2]	<0.001***	1.9 [1.2-3.0]	0.001**	2.0 [1.3-3.0]	<0.001***
Don't want to say or don't know	1.9 [0.8-4.7]		3.6 [1.8-7.6]		1.2 [0.8-1.8]		1.6 [1.0-2.4]	
Health status								
Good or fairly good health	Reference		Reference		Reference		Reference	
Moderate health	1.7 [0.9-3.0]	0.025*	1.9 [1.3-2.7]	0.002**	1.0 [0.6-1.7]	<0.001***	1.9 [1.5-2.5]	<0.001***
Poor or fairly poor health	2.9 [1.2-6.1]		2.0 [1.0-3.6]		1.8 [1.1-3.0]		2.0 [1.3-3.1]	
Principal activity								
Retired	Reference		Reference		Reference		Reference	

Working	0.6 [0.2-1.4]		0.7 [0.4-1.3]		1.4 [0.8-2.4]		0.7 [0.5-1.1]	
Student or other	1.1 [0.4-2.9]	0.250	0.9 [0.4-1.9]	0.421	2.2 [1.3-3.5]	0.003**	0.8 [0.5-1.3]	0.048*
Unemployment	1.1 [0.3-3.4]		1.1 [0.5-2.4]		1.2 [0.7-2.3]		1.3 [0.7-2.2]	
Urbanization level								
Urban	Reference		Reference		Reference		Reference	
Suburban	1.4 [0.6-3.0]	0.113	1.3 [0.8-2.2]	0.027*	1.2 [0.7-2.2]	0.729	1.0 [0.7-1.4]	0.059
Rural	2.2 [1.0-4.3]		1.9 [1.2-3.1]		1.2 [0.8-1.7]		1.5 [1.1-2.1]	
Education in health care								
No	Reference		Reference		Reference		Reference	
Yes	0.7 [0.3-1.6]	0.489	0.6 [0.3-1.1]	0.089	2.4 [1.5-4.2]	<0.001***	0.6 [0.4-0.9]	0.004**
Using internet to find health information								
No	Reference		Reference		Reference		Reference	
Yes	0.6 [0.3-1.1]	0.091	0.7 [0.5-1.2]	0.224	1.5 [1.0-2.2]	0.046*	0.8 [0.6-1.1]	0.110
Social Insurance Institution´s special reimbursement for medicines								
No	Reference		Reference		Reference		Reference	
Yes	1.2 [0.7-2.1]	0.523	1.2 [0.8-1.8]	0.303	0.9 [0.7-1.2]	0.577	1.2 [0.9-1.5]	0.253

$*** \rho < 0.001$ $** \rho < 0.01$ $* \rho < 0.05$

Table S5 Single variable analysis adjusted for age, gender and region: "Debate on vaccination issues"

	"Everyone should get vaccinated according to the national vaccination schedule"		"Vaccination does not contradict with my way of thinking"		"Anti-vaccination is a big problem in Finland"		"The media deals with vaccination and vaccine-related issues responsibly"	
Characteristic	OR $[CI]$	ρ	OR $[CI]$	ρ	OR $[CI]$	ρ	OR $[CI]$	ρ
Marital status								
In relationship	Reference		Reference		Reference		Reference	
Relationship-free	1.4 [0.9-2.1]	0.104	1.2 [0.8-1.7]	0.432	1.1 [0.9-1.3]	0.534	1.0 [0.8-1.2]	0.646
Highest education								
Tertiary education	Reference		Reference		Reference		Reference	
Upper secondary education	1.8 [1.2-2.7]	0.021*	1.5 [1.0-2.2]	0.049*	1.1 [0.9-1.3]	0.615	1.4 [1.2-1.8]	<0.001***
First degree education	1.2 [0.4-2.8]		2.0 [1.0-3.9]		0.8 [0.5-1.3]		1.8 [1.2-2.7]	
Household net income per month								
More than 5000 €	Reference		Reference		Reference		Reference	
2001-5000 €	1.6 [0.8-3.6]		1.4 [0.8-2.9]		1.1 [0.8-1.5]		1.3 [0.9-1.7]	
Up to 2000 €	2.3 [1.1-5.4]	0.020*	1.6 [0.8-3.5]	0.214	1.0 [0.7-1.4]	0.339	1.1 [0.8-1.6]	0.038*
Don't want to say or don't know	3.0 [1.4-7.1]		2.1 [1.0-4.5]		1.4 [0.9-2.0]		1.7 [1.2-2.5]	
Health status								
Good or fairly good health	Reference		Reference		Reference		Reference	
Moderate health	1.8 [1.1-2.8]	0.008**	1.2 [0.7-1.8]	0.502	0.8 [0.6-1.0]	0.079	1.5 [1.2-1.9]	0.002**
Poor or fairly poor health	2.4 [1.1-4.5]		1.5 [0.7-3.0]		0.7 [0.4-1.2]		1.5 [0.9-2.3]	
Principal activity								
Retired	Reference		Reference		Reference		Reference	
Working	1.0 [0.5-2.2]	<0.001***	0.4 [0.2-0.7]	0.019*	1.8 [1.2-2.5]	0.002**	1.4 [1.0-2.0]	0.254
Student or other	2.6 [1.1-6.0]		0.5 [0.2-1.1]		2.3 [1.5-3.4]		1.5 [1.0-2.2]	

Unemployment	2.8 [1.1-6.8]		0.7 [0.3-1.5]		2.1 [1.3-3.5]		1.5 [0.9-2.5]	
Urbanization level								
Urban	Reference		Reference		Reference		Reference	
Suburban	1.0 [0.5-1.9]	0.502	1.2 [0.6-2.0]	0.133	1.1 [0.8-1.5]	0.877	1.2 [0.8-1.6]	0.039*
Rural	1.4 [0.8-2.5]		1.7 [1.0-2.9]		1.1 [0.8-1.5]		1.5 [1.1-2.1]	
Education in health care								
No	Reference		Reference		Reference		Reference	
Yes	0.4 [0.2-0.8]	0.013*	0.8 [0.4-1.4]	0.502	0.7 [0.5-1.0]	0.045*	1.0 [0.7-1.3]	0.867
Using internet to find health information								
No	Reference		Reference		Reference		Reference	
Yes	0.6 [0.4-1.1]	0.112	0.5 [0.3-0.9]	0.018*	0.8 [0.6-1.2]	0.256	0.9 [0.7-1.2]	0.473
Social Insurance Institution´s special reimbursement for medicines								
No	Reference		Reference		Reference		Reference	
Yes	0.8 [0.5-1.3]	0.370	1.1 [0.7-1.7]	0.657	0.7 [0.6-0.9]	0.004**	1.0 [0.8-1.3]	0.932

$*** \rho < 0.001$ $** \rho < 0.01$ $* \rho < 0.05$
